# Gender differences in subliminal affective face priming: A high‐density ERP study

**DOI:** 10.1002/brb3.2060

**Published:** 2021-02-02

**Authors:** Mutsuhide Tanaka, Emi Yamada, Toshihiko Maekawa, Katsuya Ogata, Naomi Takamiya, Hisato Nakazono, Shozo Tobimatsu

**Affiliations:** ^1^ Department of Clinical Neurophysiology Neurological Institute Graduate School of Medical Sciences Kyushu University Fukuoka Japan; ^2^ Department of Occupational Therapy School of Health Science Kyushu University of Health and Welfare Nobeoka Japan

**Keywords:** emotional face processing, event‐related potentials, gender difference, N170, subliminal affective priming

## Abstract

**Introduction:**

Subliminal affective priming effects (SAPEs) refer to the phenomenon by which the presentation of an affective prime stimulus influences the subsequent affective evaluation of a target stimulus. Previous studies have reported that unconsciously processed stimuli affect behavioral performance more than consciously processed stimuli. However, the impact of SAPEs on the face‐specific N170 component is unclear. We studied how SAPEs for fearful faces affected the N170 for subsequent supraliminal target faces using event‐related potentials (ERPs).

**Methods:**

Japanese adults (*n* = 44, 20 females) participated in this study. Subliminal prime faces (neutral or fearful) were presented for 17 ms, followed by a backward mask for 283 ms and 800 ms target faces (neutral, emotionally ambiguous, or fearful). 128‐channel ERPs were recorded while participants judged the expression of target faces as neutral or fearful. Response rates and response times were also measured for assessing behavioral alterations.

**Results:**

Although the behavioral results revealed no evidence of SAPEs, we found gender‐related SAPEs in right N170 amplitude. Specifically, female participants exhibited enhanced right N170 amplitude for emotionally neutral faces primed by fearful faces, while male participants exhibited decreased N170 amplitude in fearful prime trials with fearful target faces. Male participants exhibited significant correlations between N170 amplitude and behavioral response time in the fearful prime‐neutral target condition.

**Conclusions:**

Our ERP results suggest the existence of a gender difference in target‐face processing preceded by subliminally presented face stimuli in the right occipito‐temporal region.

## Graphical abstract

We studied how subliminally presented fearful faces affected the N170 for subsequent supraliminal target faces using event‐related potentials. Female participants exhibited enhanced N170 amplitude for neutral faces primed by fearful faces, whereas male participants exhibited decreased N170 amplitude in fearful prime trials with fearful target faces. There is a remarkable gender difference in the neural processing of subliminal emotional faces.

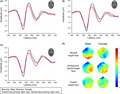

## 1 BACKGROUND

Facial information conveys the emotional status of others, making it possible to infer others’ intentions and appropriately alter subsequent social cognition and behavior. Sensitivity for face information has been reported to differ between genders, with females exhibiting a superior ability to recognize ambiguous emotional faces (i.e., 50% intensity of emotional expression) compared with males (Hoffmann et al., 2010). In addition to emotional content, greater sensitivity to human faces among females has been reported for various factors responsible for social cognition (e.g., age, faces of infants, faces of older people) (Proverbio, 2017). Electrophysiological studies using event‐related potentials (ERPs) have indicated that early face processing occurs approximately 100–200 ms poststimulus onset, reflected by the P1 (Dering et al., 2011), N170 (Bentin et al., 1996; Bruce & Young, 1986; Eimer, 2000), and P2 (Correll et al., 2006; Dennis & Chen, 2007; Schutter et al., 2004) components in occipito‐temporal regions. The N170 is reported to function as a face‐specific component, exhibiting maximal negative peak amplitude over the right occipito‐temporal region, possibly corresponding to the fusiform face area (FFA), superior temporal sulcus (STS) (Deffke et al., 2007; Nguyen & Cunnington, 2014), and the inferior occipital gyrus (IOG) (Jacques et al., 2019). The N170 component may be particularly useful for exploring assumptions of gender differences in sensitivity to emotional expressions.

In previous studies, P1 amplitude was reported to be enhanced by fearful faces significantly more than by happy or neutral faces (Del Zotto & Pegna, 2015). The posterior P2 component is also reported to reflect emotional sensitivity (Correll et al., 2006; Dennis & Chen, 2007; Schutter et al., 2004). To date, accumulating evidence has suggested that the N170 is also sensitive to emotional expression (Batty & Taylor, 2003; Hinojosa et al., 2015; Pegna et al., 2008). The N170 was found to be more sensitive to emotional facial expression stimuli compared with neutral facial expression stimuli, revealing larger amplitude, and faster latency of the N170 for emotional faces (Blau et al., 2007; Hinojosa et al., 2015). Positive expressions such as happy face stimuli evoked faster responses (i.e., faster latency) of the N170 compared with negative expressions (Batty & Taylor, 2003). Rossignol et al. (2012) reported that the N170 latency for neutral face stimuli was faster than that for fearful faces, whereas disgust and happy face stimuli elicited larger N170 amplitudes compared with fearful, angry, and neutral faces. However, another study reported larger N170 amplitudes for fearful face stimuli compared with neutral face stimuli in a range of conditions, including color face images, grayscale face images, and cropped face images with hair and ears removed (Schindler et al., 2019). Overall, differences in N170 responses among emotional faces remain to be clarified, although previous studies have provided robust evidence for sensitivity of the N170 component to emotional faces. Thus, it is currently unclear whether there is a relationship between females’ superior ability to recognize emotional expressions and the emotional sensitivity of N170.

Interestingly, the gender difference of N170 was also noted. Larger N170 amplitude for target stimuli (emotional face) compared with nontarget stimuli (emotionally neutral target) in females indicated a female advantage in allocating attention for emotional faces at an early stage (Choi et al., 2015). Females also showed greater amplitude of bilateral N170 for toddler's faces compared with adult's faces, whereas no face age effect was observed in males (Proverbio et al., 2010). In contrast, face familiarity affected N170 amplitude and latency in males, but not in females (Rostami et al., 2020). These findings suggest that the observed gender differences in the N170 component differ markedly depending on the information contained in face stimuli.

The social significance of emotional information is often assumed to be processed automatically. Although most studies define happiness, sadness, anger, disgust, surprise, and fear as basic emotions, it remains unclear whether a set of basic emotions actually exists (Celeghin et al., 2017). From an evolutionary perspective, stimuli that indicate an imminent threat, such as fearful facial expressions, should be rapidly processed to evoke extremely rapid social reactions (Costa et al., 2014). Amygdala activity reflects various categories of emotion, including sensitivity for threat‐related stimuli (Méndez‐Bértolo et al., 2016). Amygdala modulation has been observed in response to exposure to fearful face stimuli (Garvert et al., 2014; Pessoa et al., 2002; Vuilleumier et al., 2002). Threat‐related activation of the amygdala is faster and more intense when subjects are exposed to unattended or unconsciously perceived fearful stimulus (Bayle et al., 2009; Öhman et al., 2007). Lesion studies have reported that cortical blindness patients can perceive subliminal fearful face stimuli presented in the blind hemifield (“blindsight”) with enhanced cortical and subcortical responses, including amygdala and occipito‐temporal face areas (e.g., FFA) in the damaged hemifield (Morris, 2001; Pegna et al., 2005; Vuilleumier et al., 2002). Several studies have proposed that such unconscious fear processing depends on a subcortical route that typically bypasses the visual cortex. Instead, the amygdala receives threat‐related visual information via the superior colliculus and pulvinar thalamus (Öhman et al., 2007; Tamietto et al., 2012; Van den Stock et al., 2011). Thus, the subcortical pathway to the amygdala, probably including a magnocellular channel, is thought to be specialized for processing low spatial frequency information of fearful images (Diano et al., 2017). Together, these findings suggest that unconsciously perceived affective (particularly threat‐predicting) information may modulate the amygdala‐visual cortex network to regulate subsequent volitional behavior. Furthermore, several previous studies have also suggested a gender difference in amygdala activation for face stimuli (Cahill et al., 2004; Killgore & Yurgelun‐Todd, 2001; Kret & De Gelder, 2012). Thus, gender differences in the amygdala response to emotional stimuli may influence subsequent cortical activity related to facial processing, resulting in modulation of face‐related ERP components.

Amygdala modulation of occipital regions is thought to underlie subliminal affective priming, the phenomenon by which subliminal presentations of affective prime stimuli shifts subsequent affective evaluation of supraliminal target stimuli (Fazio, 2001; Murphy & Zajonc, 1993). The positive bias effect on the rating of the subsequent target stimuli was found to be primed by positive emotional images (Mohan et al., 2016). The negative bias effect has also been examined and is a phenomenon in which subliminal stimuli that would be expected to hinder social interaction (i.e., an angry face) suppress subsequent behavior (Parkinson et al., 2017). Recent neurophysiological and neuroimaging studies have used the backward masking paradigm to reveal the neural substrates underlying the processing of subliminal affective objects. Several ERP studies have suggested that ERP components, such as N2/P3 recorded from the midline site, are enhanced more by subliminally presented fearful faces than by supraliminally presented fearful faces (Axelrod et al., 2015; Kiss & Eimer, 2008; Liddell et al., 2004; Pegna et al., 2008; Vukusic et al., 2017). The larger P1 component found in the subliminal priming effect of fearful face primes is in accord with the priming effect on affective evaluation (Li et al., 2008). Occipital P2 components have also been reported to exhibit greater sensitivity for subliminal fearful or threat stimuli compared with nonfearful stimuli (Helfinstein et al., 2008; Pegna et al., 2011). Interestingly, some researchers also reported that the face‐specific N170 component also showed a marked subliminal priming effect for fearful faces (Pegna et al., 2011; Smith, 2011; Vukusic et al., 2017). Furthermore, Hietanen and Astikainen (2012) found that participants exhibited higher accuracy for target faces in an expression judgment task in the prime‐target congruent condition (positive prime‐happy target or negative prime‐sad target) and that N170 amplitude for target faces related to behavioral performance was also significantly enhanced in prime‐target congruent conditions. These findings indicate a double dissociation for subliminal versus supraliminal processing of fearful faces (Liddell et al., 2004), raising the possibility that subliminally presented fearful face stimuli may affect the perception of subsequently presented supraliminal face stimuli via subcortical visual pathways.

The possibility of gender differences in subliminal affective priming effects (SAPEs) on ERP components is an important issue that remains to be fully elucidated. Previous behavioral evidence has suggested a gender difference in affective priming, with female participants tending to respond more sensitively to affective information compared with male participants (Burton et al., 2005; Donges et al., 2012; Gohier et al., 2013). The neural basis of the greater behavioral sensitivity to emotional content exhibited by women has been examined in several ERP studies (Choi et al., 2015; Kim et al., 2013; Lee et al., 2017; Lithari et al., 2010). For example, Lee et al. (2017) reported that P100 amplitude was enhanced more when females viewed subliminal fearful face stimuli compared with males viewing the same stimuli, but failed to find a gender effect on the N170 and P2. Females also showed greater N170 amplitudes in response to target flower stimuli preceded by subliminally presented threat‐related stimuli (e.g., a man or woman with a disfigured face) compared with subliminal neutral stimuli (Kim et al., 2013). Overall, these findings indicated that subliminal threat‐related face stimuli might modulate ERP components, including the N170, especially in females, possibly depending on the type and/or duration of subliminal prime stimuli (Lee et al., 2017; Li et al., 2018; Wang & Zhang, 2016). It is possible that time windows of ERP components differ between genders if the neural basis of SAPEs differs between males and females. Therefore, we adopted microstate analysis (Cacioppo et al., 2016) instead of conventional ERP methods, and explored significant activity in the whole brain from global field power measured using high‐density electroencephalography (EEG).

The durations of subliminal stimuli reported in previous ERP studies range widely (8–50 ms), and the stimulus thresholds also differ greatly among individuals (Mitsudo et al., 2011). It has been reported that conscious perception is affected by subliminal presentation condition, and partial perception of subliminal stimuli increases subliminal priming effects on behavior (Lähteenmäki et al., 2019; Lohse & Overgaard, 2019). Lohse & Overgaard (2019) reported that participants could barely perceive subliminal stimuli at extremely short durations (8 ms), whereas durations of 25 ms enabled them to perceive stimuli. Thus, we decided to present the subliminal prime stimulus within the physical limits of the CRT monitor (i.e., 1 frame of 60 Hz = approximately 16.7 ms) aiming to present a brief glimpse of subliminal stimuli in the present study.

Finally, most previous studies of subliminal priming have used the backward masking paradigm. Thus, SAPEs to subsequent supraliminally presented target‐face stimuli remain unclear because of the superimposition of ERP responses for the prime and target faces. In previous behavioral studies, affective priming effects have been obtained with a relatively short (300 ms) prime‐target interval (stimulus onset asynchrony; SOA) (Chica et al., 2014; Fazio, 2001; Folyi et al., 2019). Therefore, the current study sought to investigate neural activity accompanying SAPEs using high‐density EEG with the subliminal priming paradigm adapted from ^t^hese ^studies^. Therefore, we presented prime facial stimuli (neutral or fearful) for 17 ms and instructed participants to judge the facial expression of supraliminally presented target faces (neutral, ambiguous‐fearful, or fearful) at 300 ms after the postprime stimulus onset.

The aim of the present study was to identify how SAPEs of fearful faces affected ERP components for subsequent supraliminal target faces and to evaluate gender differences. Previous studies employing a subliminal priming paradigm suggested that SAPEs on ERP components depended on whether a prime and a target were either affectively congruent or incongruent. In addition, some studies have reported modest or notable behavioral and neural SAPEs on emotionally ambiguous target faces primed by subliminal fearful faces (Li et al., 2008; Lu et al., 2011). Accordingly, we assumed that different emotional congruency effects (neutral prime‐neutral target or fearful prime‐fearful target) on face‐related ERP components would be found between genders (Hietanen & Astikainen, 2012). Specifically, the N170 for neutral target faces in the fearful prime condition (i.e., prime‐target emotionally incongruent condition) could be enhanced for female participants compared with male participants because of SAPEs for neutral target objects primed by fearful faces (Kim et al., 2013). Moreover, we expected to observe SAPEs for ambiguous‐fearful faces in both behavioral and ERP data, in accordance with previous reports of SAPEs for ambiguous faces (Li et al., 2008; Lu et al., 2011) and greater sensitivity for facial emotion among females (Hoffmann et al., 2010). Overall, we hypothesized that subliminally presented fearful face stimuli would negatively shift the subsequent emotional judgment of the target face, particularly for emotionally ambiguous faces in females. We also predicted that the face‐related N170 would be more affected by ambiguous‐fearful target‐face stimuli in the fearful face‐priming condition compared with those in the neutral priming condition in female participants. To achieve these objectives, we measured behavioral data and EEG during participant's facial judgment performance using high‐density EEG. We conducted microstate analysis to identify face‐related ERPs (especially the N170), time windows and regions of interest (ROIs) without any a priori hypotheses.

## 2 MATERIALS AND METHODS

### 2.1 Participants

Sample size was estimated based on the effect size of a subliminal priming study of the N170 component (*η_p_^2^ = *0.24) using G*Power (http://www.gpower.hhu.de/, RRID: SCR_013726) (Erdfelder et al., 2009). A sample size calculation indicated a total sample size of 38 (19 per group) with a power of 0.9 (1‐β) and an α error probability of 0.05.

As a result, 49 healthy Japanese adults (20–32 years old; mean age: 23.3 ± 3.3 years; 22 females) participated in this study. All participants were right‐handed, and all had normal or corrected‐to‐normal vision. In this study, we divided participants by gender because our aim was to examine gender differences in SAPEs (27 males: 24.1 ± 2.9 years, 22 females: 22.4 ± 3.6 years). None of the participants had a history of neurological or psychiatric disorders. All participants provided written informed consent. The experimental procedure was approved by the Ethics Committee at the Graduate School of Medical Science, Kyushu University (24078), and the methods were carried out in accordance with the relevant guidelines. This research was conducted in accordance with the Helsinki Declaration, revised in 1989.

### 2.2 Visual stimuli

**FIGURE 1:**
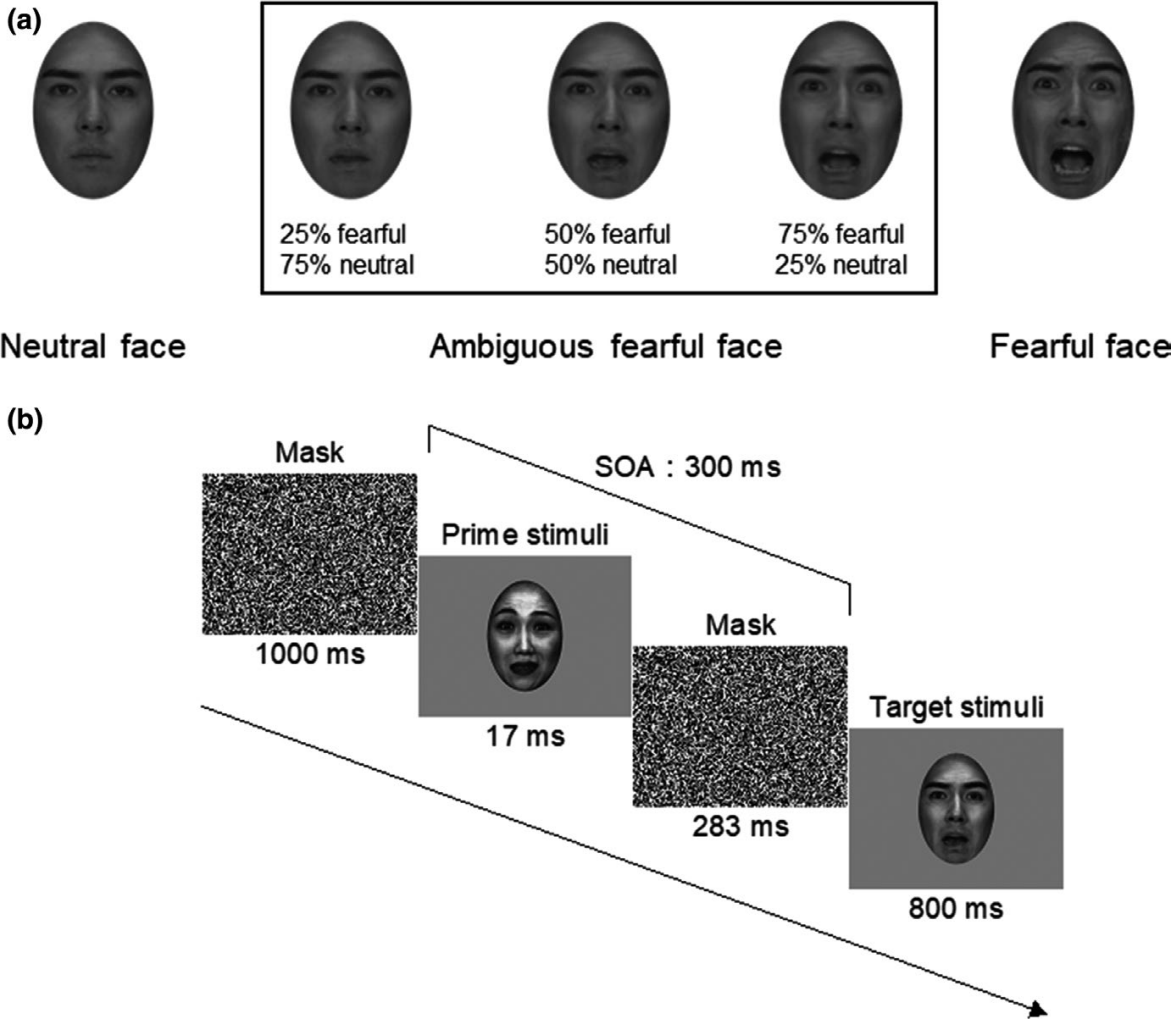
(a) Examples of the face stimuli used in the current experiment. Ambiguous‐fearful face stimuli were created by morphing neutral and fearful faces together, while keeping the same identity. Three types of 25% step‐morphed faces were used to define emotionally ambiguous‐fearful faces for each participant. (b) The experimental procedures. Each trial began with mask stimuli and a red fixation cross lasting for 1,000 ms. A prime‐face stimulus (neutral or fearful) was presented for 17 ms and immediately followed by the mask stimulus for 283 ms (SOA was 300 ms). A target‐face stimulus (neutral, ambiguous‐fearful, or fearful) then appeared for 800 ms. Participants were instructed to judge whether the target face was fearful or neutral as quickly as possible by pressing the left or right mouse button

Prime‐face stimuli consisted of grayscale photographs of four Japanese actors (two males and two females) exhibiting fearful or neutral expressions, resulting in a total of eight different prime‐face stimuli. Target‐face stimuli included fearful, ambiguous‐fearful, and neutral expressions displayed by four Japanese actors (two males and two females), who were not the actors used for the prime‐face stimuli. We used Morpher 3.1 freeware to generate a series of target‐face stimuli with ambiguous‐fearful emotional facial expressions. Ambiguous‐fearful stimuli were created by morphing together faces with fearful and neutral expressions. We generated three types of ambiguous‐face stimuli that differed in their fearful to neutral face ratios (25%, 50%, and 75% fearful faces). Thus, a total of 12 different facial stimuli were included in the ambiguous‐fearful subset of target‐face stimuli (i.e., three graded intensity levels × four identities, see Figure 1a). The original face images were taken from the ATR face database (ATR Promotions, Inc., Kyoto, Japan) (for detailed descriptions of the face database, see Supporting information S.1). All face stimuli were converted to grayscale and cropped into an oval shape to exclude all hair and nonfacial contours. Scrambled mask stimuli were generated using a MATLAB R2013a (The MathWorks Inc., Natick, MA, USA, RRID: SCR_001622) script. All images, including prime/target‐face stimuli and scrambled mask stimuli, were equated for luminance (15 cd/m^2^) and contrast (80%). All stimuli were presented on a 19‐inch CRT monitor (refresh rate: 60 Hz) at a viewing distance of 114 cm, and subtended a visual angle of 5.3° × 8° (283 × 413 pixels) (See Figure 1a).

### 2.3 Rating of ambiguous‐fearful target‐face stimuli

Participants sat in a dimly lit, sound‐attenuated, and electrically shielded room. The experiment was programmed using Presentation software (version 16.3, Neurobehavioral Systems, 2016; http://www.neurobs.com/, RRID: SCR_002521) to display the prime‐ and target‐face stimuli.

Before the ERP experiment, we used the three morphed ambiguous‐fearful face categories (25% fearful, 50% fearful, and 75% fearful) (Figure 1a) to determine the ambiguous‐fearful target face for each participant. One of three ambiguous‐fearful face categories was defined as ambiguous‐fearful face category for a participant if they judged it to be fearful approximately 50% of the time (chance level). At the beginning of each test, a scrambled mask stimulus with a red fixation cross appeared in the middle of the screen for 1,000 ms. Following a scrambled mask, one of the test faces (neutral, one of three fearful morph levels, or fearful) was displayed on the screen for 1,000 ms and then replaced by a scrambled mask. Each of the five face categories was presented 48 times in a pseudorandom order. Four face stimuli (two males and two females) were included in each face category, meaning that each facial image was presented 12 times. Participants were instructed to judge whether the target face was ‘‘neutral’’ or ‘‘fearful’’ by clicking the left or right mouse button with their right hands. Response buttons were counterbalanced across participants. We defined the ambiguous‐fearful target‐face category as stimuli for which the fearful response rate (RR) of each participant was approximately 50%. From the results of the rating procedure, the following ambiguous‐fearful face categories were adopted: 25% fearful = 10 participants (four females, mean RR = 38.9 ± 10.7%), 50% fearful = 26 participants (12 females, mean RR = 38.7 ± 7.4%), and 75% fearful = 13 participants (six females, mean RR = 29.9 ± 10.1%). After determining the ambiguous‐fearful face category for each participant, the stimuli that were ambiguous‐fearful for all participants were used as the ambiguous‐fearful face stimuli for the ERP experiment.

### 2.4 Affective subliminal priming task

We adopted the backward masking paradigm from Mitsudo et al. (2011). Each trial began with a mask stimulus and a red fixation cross lasting 1,000 ms. A prime‐face stimulus was displayed for 17 ms, immediately followed by the mask stimulus for 283 ms (SOA was 300 ms), then a target‐face stimulus was presented for 800 ms (see Figure 1b). Participants were instructed to judge whether the target face was fearful or neutral as quickly as possible by pressing the left or right mouse button with their right index finger. Participants were also instructed to fixate on the center of the display during the experiment. The target‐face stimuli did not disappear after the button response and continued to be presented until 800 ms. Because participants sustained visual attention throughout the ERP experiment, visual attention was unlikely to have caused a significant difference between the experimental conditions. The response button assignment was counterbalanced across participants. After the experiments, participants were asked if they had noticed the prime faces.

Each prime‐face stimulus subset (neutral or fearful) included four stimuli (two male actors and two female actors) and each target‐face stimulus subset (neutral, ambiguous‐fearful, or fearful) also comprised four identities. Six different trial types were formed from these prime and target pictures (i.e., neutral prime/neutral target, neutral prime/ambiguous‐fearful target, neutral prime/fearful target, fearful prime/neutral target, fearful prime/ambiguous‐fearful target, and fearful prime/fearful target). Each trial type was presented 144 times in a pseudorandom order. Thus, the total number of trials in the experiment was 864 (six trial types × 144 times). RRs and reaction times (RTs) were calculated from the behavioral data for each participant.

### 2.5 ERP recordings

ERP data were recorded while participants performed the affective subliminal priming task. Continuous ERPs were recorded using a high‐density 128‐channel system (NetAmps 200, Electrical Geodesics Inc., Eugene, OR, USA), and data were online bandpass filtered from 0.01 to 100 Hz and sampled at 1,000 Hz. ERP data were recorded continuously with the vertex electrode (Cz) as a reference and offline rereferenced to the average reference. We processed ERP data offline using Net Station 4.2 software (Electrical Geodesics Inc., Eugene, OR, USA, RRID: SCR_002453) with a 0.5–30 Hz bandpass filter, and segmented data from 100 ms before prime‐face onset to 700 ms after target‐face onset, corrected to a 100 ms prestimulus baseline. EEG epochs containing artifacts (EEG voltage exceeding 100 µV or eye movements in excess of 55 µV) were automatically rejected from further analysis.

### 2.6 Data analysis

**FIGURE 2:**
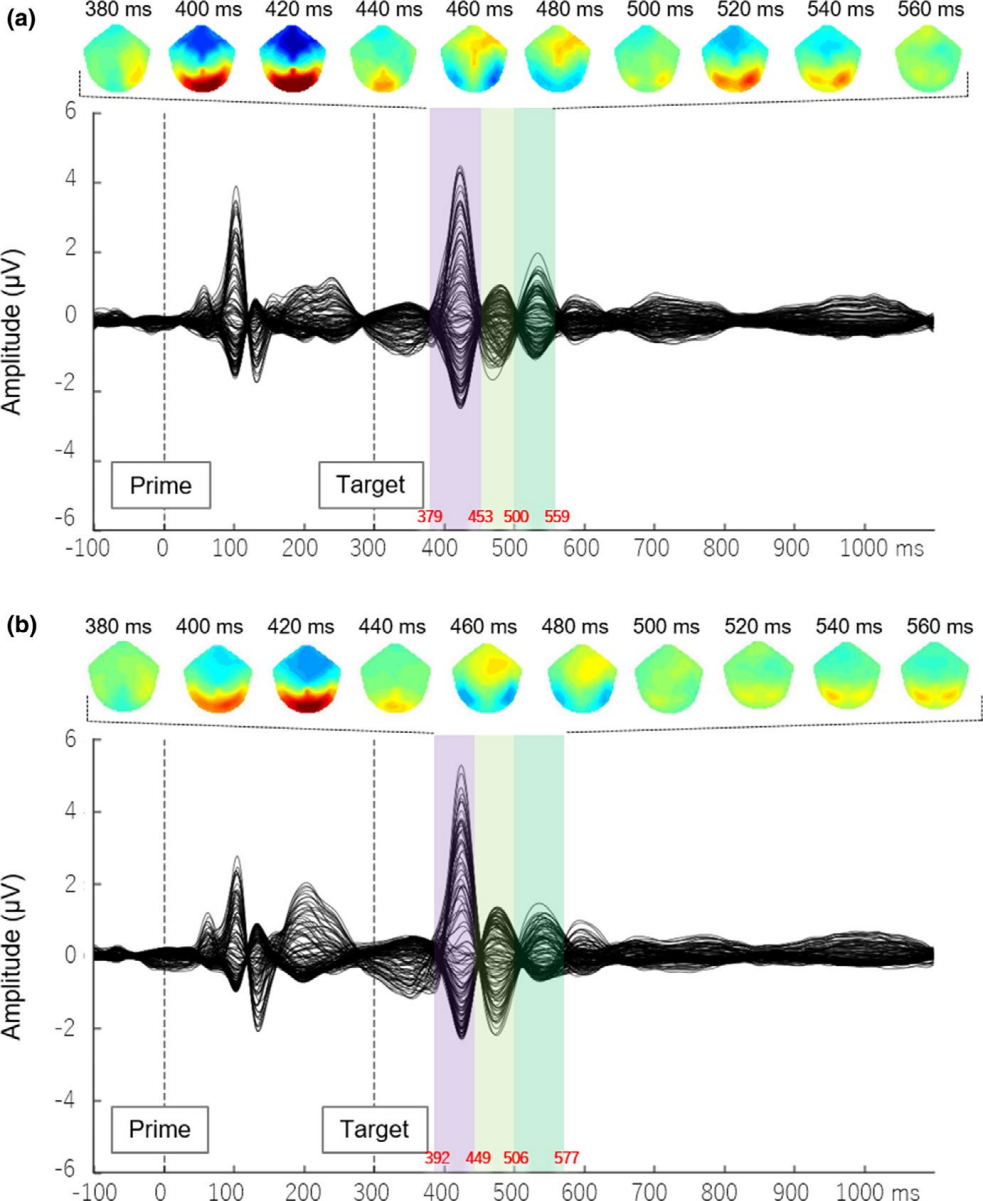
Group‐averaged ERPs during the subliminal priming task in all conditions, with waveforms from the 128 electrodes superimposed. All ERP data for each gender group (female [a] and male [b]) were summed and grand‐averaged. The ERP responses to the subliminally primed faces and target faces were clearly delineated. Note that three microstates are evident and shaded in purple, light green, and green. The red letters in the figure represented the approximate boundaries of the time window of each microstate. These microstates were further analyzed

We determined the time windows for ERP components based on butterfly plots and a microstate analysis (Cacioppo et al., 2016). First, we drew the butterfly plots by superimposing all 128 channels for all conditions and sought out salient ERP components by visual inspection (Figure 2).

**FIGURE 3:**
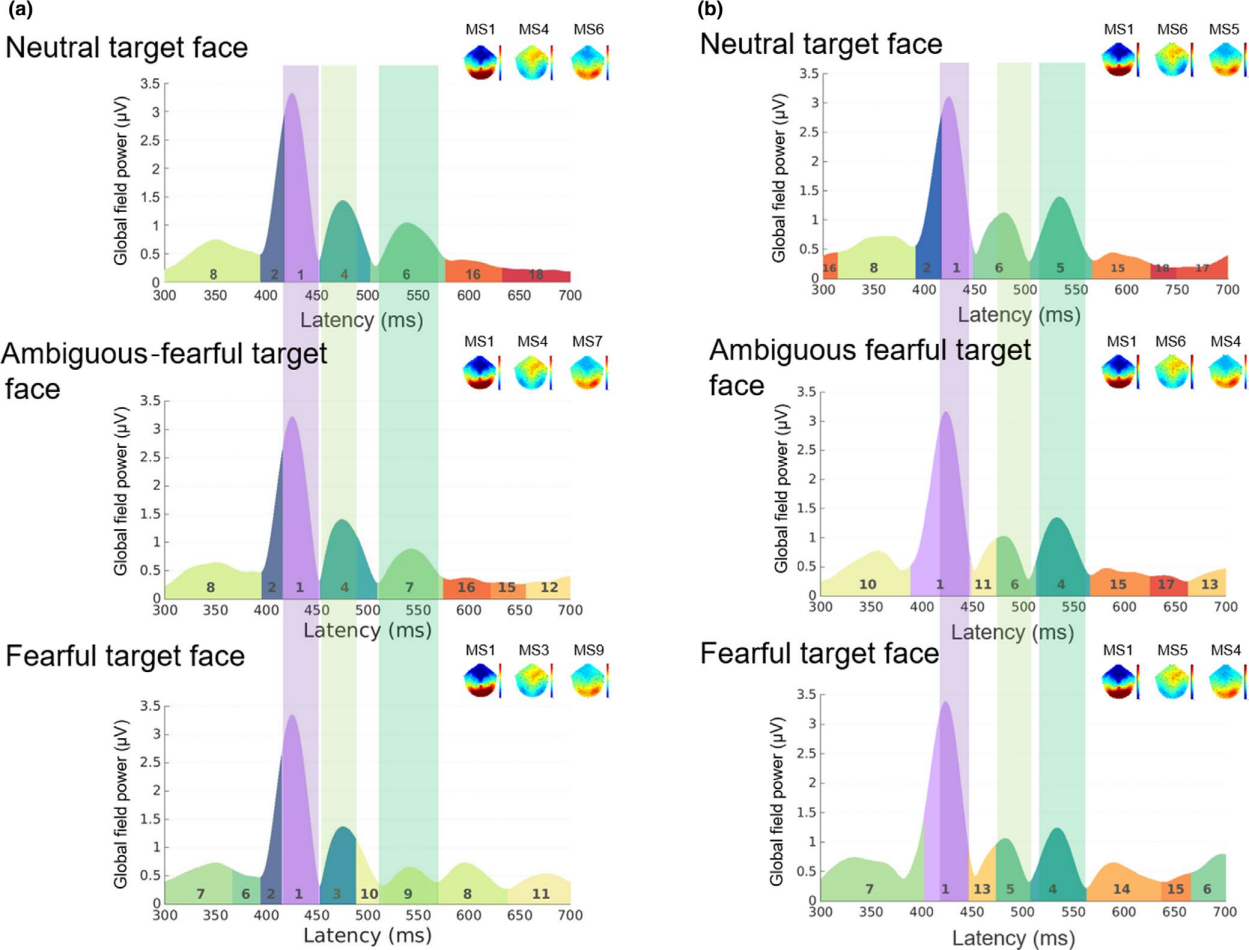
Microstate analysis outlining periods of topographic differences in the grand mean ERPs for males (A) and females (B). Fearful faces were used as the subliminal prime. Scalp topographies show microstate maps obtained from the cross‐validation procedure. The microstate maps are displayed in sequence of occurrence from left to right. Insets show the different patterns for each condition. Red and blue indicate positive and negative potential values, respectively. The onset of microstate 6 (MS6) for the ambiguous‐fearful target face, which corresponded to the N170 time window in females, was later than MS3 and MS4, which corresponded to N170 in males

Next, we applied microstate analysis to determine significant ERP components and their associated time windows (Figure 3, Supporting information Figure S1). The aim of microstate‐segment analysis is to provide information about the brain activity associated with a sequence of discrete (and nonperiodic) information‐processing operations evoked by a stimulus or task; this series of processes is composed of a series of stable brain microstates, each characterized by a particular cognitive computational operation, and a relatively stable spatial distribution of brain activity (Cacioppo et al., 2016). The brain microstate is based on the notion that the scalp potential field reflects the momentary state of global neuronal activity and that changes in the topography of this field indicate changes in the global coordination of neuronal activity over time (Michel & Koenig, 2018). Thus, the advantage of this approach is that it enables researchers to extract significant cortical responses resulting from specific experimental conditions.

Microstate‐segment analyses were carried out using Microstate Analysis Toolbox (MST) (Poulsen et al., 2018) from the EEGLAB (http://sccn.ucsd.edu/eeglab/index.html, RRID:SCR_007292) toolbox (Delorme & Makeig, 2004). All ERP data from the six conditions (male or female × six stimuli) were added together and grand‐averaged. They were then segmented into three and 20 microstate classes using a K‐means algorithm with 50 random initializations for clustering. Generally, this method requires presetting the number of clusters. We found 16 to 20 large or small envelopes in butterfly plots of each condition. The time scale contained at least three conditions (pre, prime, and target). Therefore, we determined the number of clusters as 3–20. Finally, we selected microstates using three criteria: global explained variance (GEV), cross‐validation (CV), and the Krzanowski‐Lai (K‐L) criterion. In our data, the suitable number of segments for explaining all epochs was 18. Accordingly, we fit the microstate prototypes back to all grand‐averaged data. This procedure enabled us to label the EEG epochs with the microstate prototypes.

### 2.7 Statistical analysis

For SAPEs, RRs, and RTs were analyzed with a 2 × 3 × 2 repeated‐measures analysis of variance (ANOVA) with prime‐face category (neutral or fearful) and target‐face category (neutral, ambiguous‐fearful, or fearful) as within‐participant factors, and gender (female or male) as the between‐participant factor. ERP amplitudes and latencies were analyzed using a 2 × 3 × 2 × 2 repeated‐measures ANOVA with prime‐face category (neutral or fearful), target‐face category (neutral, ambiguous‐fearful, or fearful), and laterality (left or right ROI) as within‐participant factors, gender (female or male) as the between participants factor. All analyses were conducted with SPSS (version 26; IBM Corp, USA, RRID: SCR_019096). All significant *p*‐values were corrected by Greenhouse–Geisser correction, and Šidák correction was used for multiple comparisons.

Correlation analyses were also conducted to evaluate the correlation between behavioral and ERP results. We computed Pearson's correlation between behavioral data and amplitudes of each ERP component. Because our aim was to verify SAPEs for cortical responses and behavioral performance, correlation analysis was performed only if there was a significant main effect of prime‐face category or prime face‐related interaction revealed by the mixed‐design ANOVA for ERP amplitudes.

## 3 RESULTS

### 3.1 Behavioral results

Data from five participants (three males and two females) were discarded because they contained inappropriate behavioral responses (i.e., mean RR or mean RT for the target‐face stimuli exceeded more than three times the inter‐quartile range). Thus, the final analysis included data from 44 participants (24 males: 24.0 ± 3.0 years, 20 females: 24.0 ± 3.7 years). No significant difference in age was found between two groups (*t* [42] = 1.43, *p = *.161). After the experiment, all participants reported that they were completely unaware that prime‐face stimuli had been presented. Behavioral results indicated that mean RR for ambiguous‐fearful target faces was 33.8% ± 3.1%. RR > 50% indicated a bias toward fearful stimuli.

**FIGURE 4:**
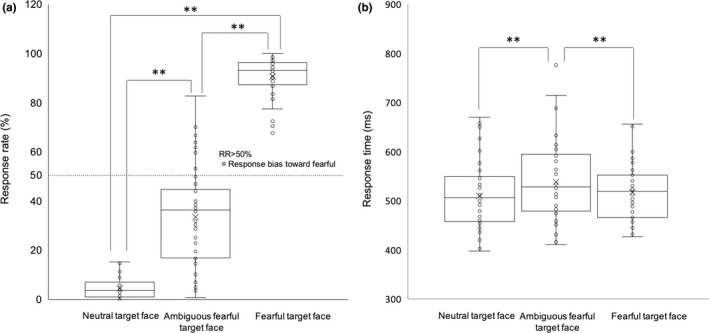
Behavioral results for response rates (RRs) and reaction times (RTs). RRs for the fearful face‐priming condition were slightly higher than those for the neutral face‐priming condition (a). RRs for fearful target‐face stimuli were significantly higher than those for neutral and ambiguous‐fearful target faces (b). RRs for ambiguous‐fearful target faces were also significantly higher than those for neutral target faces. Gender differences were not significant. Reaction times (RTs) for the ambiguous‐fearful target‐face stimuli were significantly higher than those for neutral and fearful target faces (c). “×” in the box plots represent average values. *p < .05, **p < .01

Table 1 summarizes the results of the three‐way ANOVA (prime × target ×gender) for RRs and RTs. We found a significant main effect of target face (Table 1). Post hoc analysis revealed significantly higher RRs for fearful target‐face stimuli than for neutral and ambiguous‐fearful target‐face stimuli (fearful target‐face vs. neutral target‐face = 90.87 ± 1.18% vs. 4.57 ± 0.62%, *p* < .001, fearful target‐face vs. ambiguous‐fearful target‐face = 90.87 ± 1.18% vs. 34.53 ± 3.06%, *F*[2, 41] = 2,668.20, *p* < .001, *η_p_^2^ = *0.99). RRs for ambiguous‐fearful target‐face stimuli were also significantly higher than those for neutral target‐face stimuli (ambiguous‐fearful target‐face vs. neutral target‐face = 34.53 ± 3.06% vs. 4.57 ± 0.62%, *F*[2, 41] = 2,668.20, *p* < .001, *η_p_^2^ = *0.99) (Figure 4a). We observed a marginally significant main effect of prime face (*p = *.065). However, we did not find any significant main effect of gender or any significant interactions between prime face, target face, or gender (Table 1).

**TABLE 1:** Three‐way repeated‐measures ANOVA results for the behavioral data

Factor	RR				RT			
	*df*	F	*η_p_* ^2^	*p*	*df*	F	*η_p_* ^2^	*p*
Prime face	1, 42	3.59	0.079	.065	1, 42	0.01	0.000	.943
Target face	1.30, 54.62	734.34	0.946	**<.001**	1.41, 61.75	11.83	0.220	**<.001**
Gender	1, 42	2.22	0.050	.144	1, 42	0.01	0.000	.911
Prime face × Target face	1.71, 71.60	0.44	0.010	.614	2, 84	0.60	0.014	.550
Prime face × Gender	1, 42	1.25	0.029	.271	1, 42	0.14	0.003	.708
Target face × Gender	1.30, 54.62	2.86	0.064	.086	1.47, 61.75	1.84	0.042	.177
Prime face × Target face × Gender	1.71, 71.60	0.11	0.003	.882	2, 84	0.56	0.013	.574

Note

Prime faces comprised fearful and neutral faces displayed by four Japanese actors (two males and two females). Target faces comprised fearful, ambiguous‐fearful, and neutral faces displayed by four Japanese actors (two males and two females), who were not the same actors used for the Prime faces.

Abbreviations: *df*, degree of freedom, RR, the fearful response rate; RT, reaction time in all Tables.

Bold font indicates that differences were statistically significant in Table 1 and Table 2.

The three‐way ANOVA (prime × target ×gender) for RTs revealed a significant main effect of target face (Table 1). Post hoc analysis revealed that RTs for the ambiguous‐fearful target faces were significantly longer than those for neutral faces and fearful faces (ambiguous‐fearful target‐face vs. neutral target‐face = 538.65 ± 12.54 ms vs. 511.23 ± 11.08 ms, ambiguous‐fearful target‐face vs. fearful target‐face = 538.65 ± 12.54 ms vs. 516.60 ± 8.62 ms, *F*[2,41] = 26.00, *p* < .001, *η_p_^2^ = *0.56) (Figure 4b). No other main effects or interactions were significant.

### 3.2 ERP results

#### 3.2.1 Microstate analysis

Figure 2 shows butterfly plots for each group. We found three major components within an approximately 400‐ to 580‐ms time window (100–280 ms after target‐face onset) for male and female data sets. Interestingly, we observed temporal differences among these components between the female and male groups. The microstate analysis (Figure 3, Supporting information Figure S1) revealed three microstates between 418 and 659 ms (118–359 ms after target‐face onset) for each prime × target condition. Microstate topography suggested that each microstate responded strongly among regions near the occipito‐temporal electrodes. The first microstate overlapped between 417 and 445 (117–145) ms in females and between 419 and 447 (119–147) ms in males. The second microstate overlapped between 473 and 503 (173–203) ms in females and at 444–488 (144–188) ms in males. The third microstate overlapped between 511 and 561 (211–261) ms in females and between 515 and 559 (215–259) ms in males. We used these time windows to determine the conventional ERP components: P1, N170, and P2. Peak amplitudes and latencies for the ERP components were calculated at clusters of electrodes, corresponding to specific ROIs. Because nonindependent selective analysis (i.e., the use of the same data for selection and selective analysis simultaneously) would result in distorted descriptive statistics and invalid statistical inference (Kriegeskorte et al., 2009), inflating type 1 error (Brooks et al., 2017), we selected electrodes for ROIs according to previous studies. Specifically, P1 (Rossion & Caharel, 2011) and P2 (Rossignol et al., 2013) were measured from electrodes around O1 and O2 (Rossion & Caharel, 2011). N170 was measured from electrodes around T5 and T6 (Hinojosa et al., 2015) (for detailed descriptions of ROI definitions, see section Supporting information S.2, Figure S2).

#### 3.2.2 P1 component

The results of the four‐way ANOVA (prime × target ×laterality × gender) are shown in Table 2. We found a significant main effect of target face on P1 amplitude, suggesting that P1 amplitude for fearful target faces is significantly greater than that for neutral and ambiguous‐fearful target faces (fearful vs. neutral vs. ambiguous‐fearful target face = 4.70 ± 0.24 µV vs. 4.41 ± 0.24 µV vs. 4.41 ± 0.24 µV, *F*[2, 41] = 25.58, *p* < .001, *η_p_^2^ = *0.56). We also found a significant main effect of Laterality, with P1 amplitude being significantly larger in the right occipital ROI than in the left ROI (Right vs. Left = 4.76 ± 0.25 µV vs. 4.27 ± 0.26 µV, *F*[1, 42] = 6.66, *p = *.013, *η_p_^2^ = *0.14). ANOVA also revealed a significant interaction of target face × laterality. P1 amplitude for fearful target faces was significantly greater than that of neutral and ambiguous‐fearful target faces in both ROIs (left ROI: *F*[2, 41] = 16.50, *p* < .001, *η_p_^2^ = *0.45, right ROI: *F*(2, 41) = 24.23, *p* < .001, *η_p_^2^* = 0.54) (for the time‐course representation of the ERPs, see Supporting information Figure S3).

**TABLE 2:** Four‐way repeated‐measures ANOVA results for the P1, N170, and P2 components

Factor		Amplitude			
		*df*	F	*η_p_* ^2^	*p*
P1					
	Prime face	1, 42	1.76	0.040	.191
	Target face	2, 84	19.74	0.320	**<.001**
	Laterality	1, 42	6.66	0.137	.013
	Gender	1, 42	2.17	0.049	.148
	Target face × Laterality	1.70, 71.59	3.73	0.081	**.035**
N170					
	Prime face	1, 42	0.19	0.005	.663
	Target face	1.73, 72.45	0.34	0.008	.681
	Laterality	1, 42	1.07	0.025	.308
	Gender	1, 42	16.19	0.278	**<.001**
	Prime face × Laterality ×Gender	1, 42	9.87	0.190	**<.001**
	Prime face × Target face × Laterality ×Gender	2, 84	3.50	0.077	**.035**
P2					
	Prime face	1, 42	2.32	0.052	.135
	Target face	1.62, 68.22	7.63	0.154	**.002**
	Laterality	1, 42	0.21	0.005	.652
	Gender	1, 42	0.10	0.002	.751
	Prime × Laterality	1, 42	4.52	0.097	**.039**
	Target face × Laterality Gender	1.61, 67.81	4.27	0.092	**.025**

Factor		Latency			
		*df*	F	*η_p_* ^2^	*p*
P1					
	Prime face	1, 42	0.01	0.000	.922
	Target face	2, 84	1.68	0.038	.196
	Laterality	1, 42	5.18	0.110	**.028**
	Gender	1, 42	1.49	0.034	.229
N170					
	Prime face	1, 42	5.32	0.112	**.026**
	Target face	2, 84	0.24	0.006	.784
	Laterality	1, 42	1.59	0.037	.214
	Gender	1, 42	5.38	0.114	**.025**
	Prime face × Target face × Gender	2, 84	3.17	0.070	**.047**
P2					
	Prime face	1, 42	2.20	0.050	.146
	Target face	2, 84	0.06	0.001	.943
	Laterality	1, 42	0.25	0.006	.620
	Gender	1, 42	1.74	0.040	.194

A four‐way ANOVA (prime × target × laterality × gender) of P1 latencies revealed a significant main effect of laterality (Table 2) such that P1 latency in the right ROI was significantly shorter than in the left ROI (Right vs. Left = 425.37 ± 0.92 ms vs. 426.58 ± 0.94 ms, *F*(1, 42) = 5.18, *p = *.028, *η_p_^2^* = 0.11). We found no significant main effects for the prime conditions.

#### 3.2.3 N170 component

**FIGURE 5:**
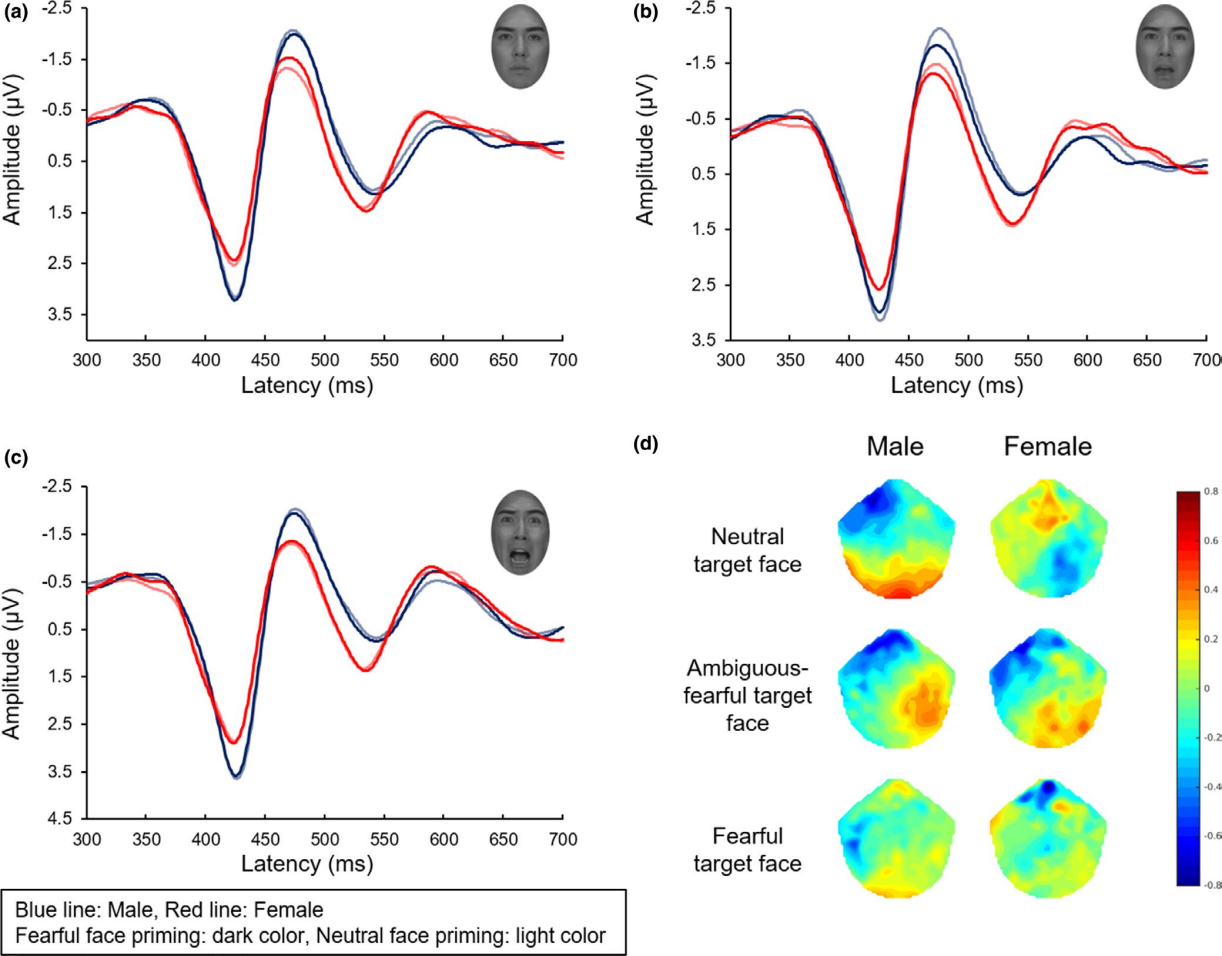
Grand‐averaged waveforms for the N170 in the right ROI of male and female participants and their scalp topographies. (a) Grand average ERPs to neutral target faces, (b) ambiguous‐fearful target faces, and (c) fearful target faces. (d) Scalp topographies for each target‐face type were calculated by subtracting the N170 response in the neutral face prime condition from that in the fearful face prime condition. The N170 amplitude for ambiguous‐fearful target‐face stimuli was smaller in the fearful prime condition compared with the neutral prime condition in both groups (b, d). N170 amplitudes in female participants differed from those in males for both primed conditions (a, c) as did the scalp topography (d). In female participants, N170 amplitudes for neutral target faces in the fearful prime condition were larger than those in the neutral prime condition. Meanwhile, in male participants, N170 amplitudes for neutral target faces and fearful target faces in the fearful prime condition were slightly smaller than those in the neutral prime condition. For graphical presentation, ERP waveforms are shown 300 ms after the prime stimuli onsets. The y‐axis ranges from − 2.3 to 3.0 µV for better viewing of the N170 waveforms

Although no significant difference in prime conditions or target‐face categories was evident in the grand‐averaged waveforms (or the time‐course representation of the ERPs, see Supporting information Figure S3), a gender difference in SAPEs was found in the grand‐averaged waveforms in the right ROI (Figure 5). Specifically, the N170 amplitude for ambiguous‐fearful target‐face stimuli was smaller in the fearful prime condition compared with the neutral prime condition in both groups (Figure 5b,d). The N170 amplitude for neutral target‐face stimuli was larger in the fearful prime condition compared with the neutral prime condition in female participants (Figure 5a,d). Moreover, the N170 amplitude for fearful target‐face stimuli primed by fearful face stimuli was slightly smaller than N170 amplitude for fearful target‐face stimuli primed by neutral face stimuli in male participants (Figure 5c,d).

**FIGURE 6:**
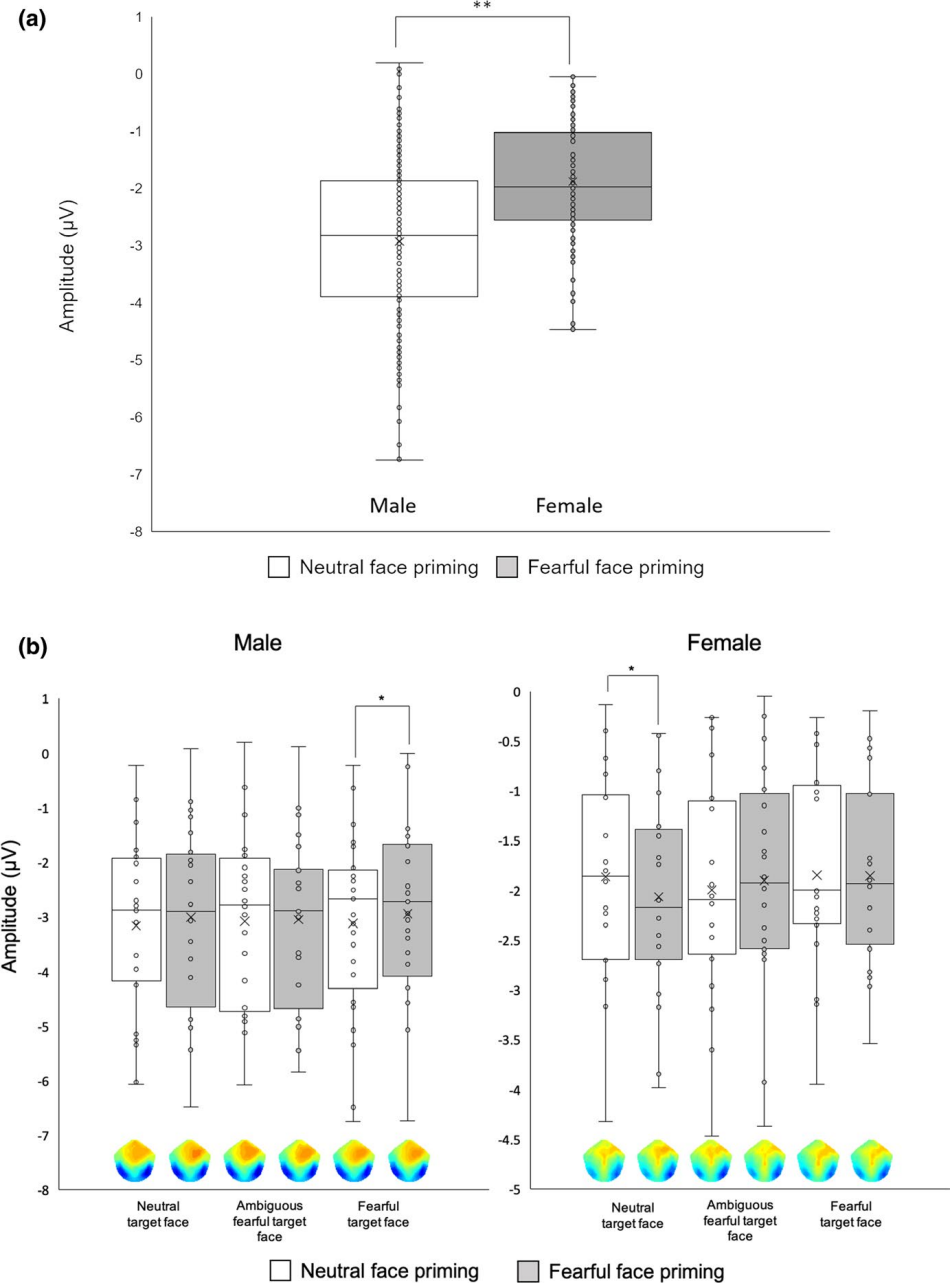
ERP results for N170 amplitudes. N170 amplitudes of male participants were significantly larger than those of female participants (a). N170 amplitudes for neutral target faces in the fearful prime‐face condition were significantly larger than those in the neutral prime‐face condition in the right ROI in females (b). Scalp topographies below the bar plots show peak amplitudes of the N170 for each experimental condition. Male participants demonstrated smaller N170 amplitudes for fearful target faces in the neutral prime condition than those in the fearful prime condition in the right ROI (b). “×” in the box plots represent average values. *p < .05, **p < .01

Four‐way ANOVA (prime × target × laterality × gender) calculated for N170 amplitude revealed a significant main effect of Gender (Table 2). A direct comparison between genders revealed larger N170 amplitude in males compared with females (Males vs. Females = −2.79 ± 0.22 µV vs. −1.50 ± 0.24 µV, *F*[1, 42] = 16.20, *p* < .001, *η_p_^2^ = *0.28) (Figure 6a). We also found a significant interaction of prime face × laterality × gender (Table 2), with N170 amplitude in the fearful prime‐face condition being significantly larger than that in neutral prime‐face condition in the right occipital ROI for males (fearful face prime vs. neutral face prime = −2.69 ± 0.25 µV vs. −2.62 ± 0.24 µV, *F*[1, 42] = 6.01, *p* = .019, *η_p_^2^ = *0.13). Furthermore, ANOVA revealed a significant interaction of prime face × target face × laterality × gender (Table 2). A post hoc test revealed gender differences of SAPEs in prime/target congruent/incongruent conditions. The N170 amplitude for neutral target faces in the fearful prime‐face condition was also significantly larger than that in the neutral prime‐face condition in the right ROI in females (fearful prime face vs. neutral prime face = −1.70 ± 0.31 µV vs. −1.47 ± 0.29 µV, *F*[1, 42] = 6.63, *p* = .014, *η_p_^2^ = *0.14) (Figures 5a, 5d and 6b). Conversely, the N170 amplitude for the fearful target faces in the fearful prime‐face condition was significantly smaller than that in the neutral prime‐face condition in the right ROI in males (fearful prime face vs. neutral prime face = −2.79 ± 0.27 µV vs. −3.00 ± 0.23 µV, *F*[1, 42] = 6.08, *p* = .02, *η_p_^2^ = *0.13) (Figures 5c,d and 6b). We did not observe any main effects of prime condition, target face, or laterality. Furthermore, we conducted mixed‐designed ANOVA excluding “laterality,” because the visual inspection of scalp topographies and grand‐averaged waveforms suggested a possible gender difference in the lateralization of the distribution. The results revealed a main effect of Gender (*F*[1, 42] = 21.72, *p* < .001, *η_p_^2^ = *0.20) with larger N170 amplitude in males relative to females (males vs. females = −2.64 ± 0.14 µV vs. −1.67 ± 0.15 µV, *p* < .001). We also found a significant interaction between prime face × gender (*F*[1, 86] = 6.61, *p* = .012, *η_p_^2^ = *0.07) with males exhibiting a significantly larger N170 amplitude than females in both prime‐face conditions (fearful prime‐face condition: males vs. females = −2.49 ± 0.15 µV vs. −1.84 ± 0.16 µV, *F*[1, 86] = 8.57, *p* < .001, *η_p_^2^ = *0.09, neutral prime‐face condition: males vs. females = −2.80 ± 0.18 µV vs. −1.51 ± 0.19 µV, *F*[1, 86] = 24.39, *p* < .001, *η_p_^2^ = *0.22).

Four‐way ANOVA (prime × target ×laterality × prime) calculated for N170 latency revealed a significant main effect of gender (Table 2), suggesting that the N170 latency in males was significantly shorter than that in females (male vs. female = 472.39 ± 2.01 vs. 479.30 ± 2.20, *p = *.025). The main effect of prime faces was also significant (Table 2), suggesting that the N170 latency in the fearful prime‐face condition was significantly shorter than that in the neutral prime‐face condition (fearful prime face vs. neutral prime face = 475.54 ± 1.50 vs. 476.15 ± 1.49, *F*[1, 42] = 5.32, *p* = .026, *η_p_^2^ = *0.11). We also found a significant interaction between prime face, target face, and gender (Table 2), with the N170 latency for neutral target face in fearful prime‐face condition being significantly shorter than that in neutral prime‐face condition in females (fearful face prime vs. neutral face prime = 478.1 ± 2.18 vs. 480.25 ± 2.40, *F*[1, 42] = 5.99, *p* = .019, *η_p_^2^ = *0.13) (Table 2).

#### 3.2.4 P2 component

A four‐way ANOVA (prime × target ×laterality × gender) examining P2 amplitude revealed a significant main effect of target face (Table 2). A direct comparison between target‐face categories revealed that P2 amplitudes were higher for neutral target faces than for fearful target faces (1.72 ± 0.20 µV vs. 1.53 ± 0.19 µV, *F*[2, 41] = 6.42, *p* < .001, *η_p_^2^ = *0.24). ANOVA also revealed a significant prime face × laterality interaction (Table 2). P2 amplitude in the fearful prime‐face condition was significantly larger than that in the neutral face prime condition in the right ROI (1.70 ± 0.20 vs. 1.61 ± 0.21 µ V, *F*[1, 42] = 5.56, *p* = .023, *η_p_^2^ = *0.12). Furthermore, there was a significant interaction between target face, laterality, and gender (Table 2). Further analysis revealed that P2 amplitude for neutral target faces in the left ROI was significantly larger than ambiguous‐fearful and fearful target faces, only for males (neutral target face vs. ambiguous‐fearful target face vs. fearful target face = 1.88 ± 0.26 µV vs. 1.71 ± 0.26 µV vs. 1.49 ± 0.26 µV, *F*[2, 41] = 14.01, *p* < .001, *η_p_^2^ = *0.41) (for a time‐course representation of the ERPs, see Supporting information Figure S3). We found no main effects or interactions for P2 latency (Table 2).

#### 3.2.5 Correlations between amplitudes of each ERP component and behavioral data

The results of mixed‐design ANOVA for quantitative ERP data revealed a significant interaction of prime face × target face × laterality ×gender for N170 amplitude and prime face × laterality for P2 amplitude. Therefore, we conducted a correlation analysis between N170 amplitude and RR/RT for all combinations of four factors, whereas correlation analyses between P2 amplitude and behavioral data were performed with combinations of two intra‐participants factors (prime face and laterality).

In male participants, N170 amplitude for neutral and fearful target faces in the fearful prime condition in the right ROI was only positively correlated with RT (neutral target face: *r = *0.430, *p* = .036, fearful target face: *r* = 0.468, *p = *.021). In females, however, we found no significant correlations between N170 amplitude and behavioral data. There was also no significant correlation between P2 amplitude and behavioral data (Table 3 and 4).

**TABLE 3:** Correlation coefficients (r) between behavioral data and amplitudes of N170 components in the fearful prime‐face condition

Behavioral data	Group	Laterality	Fearful prime		
			Neutral target	Ambiguous‐fearful target	Fearful target
RR	Males	Left	−0.058	−0.031	−0.115
		Right	0.076	−0.033	−0.051
	Females	Left	0.038	−0.021	−0.375
		Right	0.253	0.021	−0.075
RT	Males	Left	−0.078	0.017	0.033
		Right	0.430*	0.351	0.468*
	Females	Left	−0.055	−0.067	0.107
		Right	0.150	0.072	−0.137

Notes

^*^ *p* < .05.

**TABLE 4:** Correlations between behavioral data and amplitudes of N170 components in the neutral prime‐face condition

Behavioral data	Group	Laterality	Neutral prime		
			Neutral target	Ambiguous‐fearful target	Fearful target
RR	Males	Left	−0.138	−0.042	−0.242
		Right	−0.028	0.006	−0.065
	Females	Left	−0.121	−0.156	−0.422
		Right	−0.085	0.032	−0.122
RT	Males	Left	−0.127	−0.061	−0.093
		Right	0.398	0.359	0.381
	Females	Left	−0.081	−0.051	−0.180
		Right	−0.024	0.026	−0.048

## 4 DISCUSSION

In the current study, we explored the neural correlates of SAPEs in terms of behavioral performance and high‐density ERPs. We compared ERPs in response to three types of target‐face stimuli (neutral, ambiguous‐fearful, and fearful) after participants were primed by neutral and fearful face stimuli that were presented for only 17 ms, and were thus subliminal. Interestingly, we found gender differences in SAPEs for the face‐specific N170 component in the Right Hemisphere. Female participants exhibited larger N170 amplitude for neutral faces primed by fearful faces compared with those primed by neutral faces, whereas male participants showed smaller N170 amplitudes for fearful target faces primed by fearful faces compared with those primed by neutral faces. Correlation analysis revealed that the male group showed a significant correlation between N170 amplitude and behavioral RT in the fearful prime‐neutral target condition. Moreover, the male group showed an emotional congruency (fearful prime‐fearful target) effect in right N170 amplitude. These results suggested that SAPEs of fearful faces occurred for neutral target‐face stimuli in females and males, but in different ways: Females showed enhanced N170 amplitude whereas males showed a positive correlation between N170 amplitude and behavioral performance. Our results imply the possibility that the N170 component reflects the neural basis of the SAPE, and its impact on behavioral performance may differ between genders. Several previous studies have reported gender differences in the N170/M170 component under supraliminal conditions (Choi et al., 2015; Proverbio et al., 2010; Rostami et al., 2020; Tiedt et al., 2013). However, no previous studies have demonstrated gender differences in SAPEs using ERPs, including face‐specific N170 components. Therefore, the current study provides the first evidence of a gender difference in target‐face processing preceded by subliminally presented face stimuli in the right occipito‐temporal regions.

Affective priming typically leads to shorter reaction times when judging targets, and emotional judgments of neutral objects are affected by briefly presented emotional prime faces (Fazio, 2001; Murphy & Zajonc, 1993; Winkielman et al., 2005). Some studies have reported gender differences in the affective priming effect. Gohier et al. (2013) reported greater sensitivity to negative emotional prime‐face stimuli in females than in males, whereas Donges et al. (2012) demonstrated stronger positive priming in females than in males. According to previous studies, we expected that increased RR and shortened RT would occur in response to ambiguous‐fearful faces primed by fearful faces in females. However, we did not observe significant SAPEs in the behavioral results. Rather, we observed longer RTs for ambiguous‐fearful target faces than for neutral or fearful target faces. It remains to be confirmed whether this finding reflects difficulty of the emotional judgment or a negative effect of priming.

Until now, the existence of SAPEs has been controversial, and some studies have failed to demonstrate an affective priming effect (Andrews et al., 2011; Comesaña et al., 2013). This inconsistency in previous studies could be explained by the prime stimulus (schematic images or emotional words) or task used (forced‐alternative task). The presentation time of the prime stimuli can be critical because previous behavioral studies adopted longer presentation times than the present study (20–250 ms). Accordingly, the subliminal priming effect may not be elicited if participants are unaware of the subliminal stimuli at all (conversely, partial awareness could elicit the priming effect) (Lähteenmäki et al., 2019; Lohse & Overgaard, 2019). In this study, all participants reported that they were completely unaware of prime‐face stimuli, resulting in no SAPEs in the behavioral data. In contrast, N170 amplitude was positively correlated with RTs in male participants. This suggests that early neural processing of target faces might affect how rapidly they can be judged and that the N170 component could reflect SAPEs more sensitively than behavioral performance, as discussed in more detail below.

Consistent with previous studies, our results showed the sensitivity of P1 and P2 components for emotion (i.e., greater P1 amplitudes for fearful target faces than that for neutral and ambiguous‐fearful target faces, but smaller P2 amplitudes for fearful target than for neutral target faces). Other studies also reported prime‐target emotional incongruency effects on P1 (Meeren et al., 2005; Werheid et al., 2005) and a subliminal negative affective priming effect on P2 (Bernat et al., 2001; Elgendi et al., 2018). Although we found enhanced P2 amplitudes by fearful prime faces in the right hemisphere, irrespective of target‐face expressions, the present study failed to demonstrate SAPEs in the P1 and P2 components. Most previous studies adopted the backward masking paradigm and focused on analyzing subliminal effects on prime stimuli, whereas the current study aimed to analyze SAPEs of subsequent target stimuli. Thus, our data suggested that SAPEs did not affect P1 and P2, but did affect the N170 component.

The present study demonstrated that the right posterior N170 showed salient SAPEs, with shorter latencies for neutral target faces in the fearful face prime conditions than those in the neutral face prime conditions. Moreover, females exhibited greater amplitudes for neutral target faces in the fearful face prime conditions than those in the neutral face prime conditions. Meanwhile, male participants in the present study showed decreased right N170 amplitudes in fearful prime/fearful target conditions. Behavioral results also demonstrated significant positive correlations between RTs and N170 amplitude for neutral and fearful target faces primed by fearful faces. The reasons for gender differences in SAPEs for fearful target faces may be explained by prime‐target congruency/incongruency effects. That is, emotional information of consciously perceived stimuli, incongruent with unconsciously predicted emotional information (i.e., fearful prime‐neutral target), enhanced the N170 response in females, while male participants exhibited significant emotional repetition suppression (i.e., fearful prime‐fearful target). Previous studies reported that amygdala activity was decreased by repetition of negative stimuli (Britton et al., 2008; Ishai et al., 2004; Phan et al., 2003). Furthermore, another study suggested that gender differences in brain activity depended on the emotional categories or presentation conditions of emotional stimuli (Kret & De Gelder, 2012). Thus, it is possible that our data revealed a different emotional congruency/incongruency effect on the N170 because of differences in neural processing between genders.

Previous ERP studies reported a prime‐target emotional congruency/incongruency effect on the N170 (Diéguez‐Risco et al., 2015; Hietanen & Astikainen, 2012). An incongruency effect on the N400 component by various modalities has also been reported (Kutas & Federmeier, 2011). However, these previous studies used nonface target stimuli or supraliminally presented prime faces and reported no gender differences. A differential effect direction in previous studies may indicate differences in the processing mechanisms underlying contextual effects under different task conditions, stimulus types, and stimulus presentation methods. However, no previous studies have utilized the subliminal priming paradigm used in the current study. Because the subliminal prime stimuli used in present study could not be subjectively perceived by participants, they were perfectly unconscious prime stimuli. Therefore, the present findings provide the first evidence of gender differences in the emotional congruency/incongruency effect of SAPEs on the N170.

The N170 component is generated in the FFA, while the STS is considered to be specialized for visual processing and encoding of high level features such as structural information contained within faces (Bentin et al., 1996; Bruce & Young, 1986; Itier & Taylor, 2002; Rossion, 2014). Although some researchers (Eimer & Holmes, 2002; Kiss & Eimer, 2008) have claimed that the N170 is relatively insensitive to emotion, several studies have observed the sensitivity of this component to emotional facial expressions (Aguado et al., 2013; Batty & Taylor, 2003; Hinojosa et al., 2015).

Based on these controversial findings, many previous studies have examined the emotion‐based modulation of the N170 component by looking for effects related to the emotional valence of prime stimuli (Pegna et al., 2011; Smith, 2011; Vukusic et al., 2017). ERP studies using the affective priming paradigm have reported enhanced N170 amplitude for target stimuli when the emotional valence of prime visual stimuli or scenes is congruent with that of target stimuli (Hietanen & Astikainen, 2012; Righart & De Gelder, 2006, 2008). Although negative evidence for affective priming effects on the N170 component has been reported (Krombholz et al., 2007; Werheid et al., 2005), one explanation for this discrepancy between the current findings and previous studies could be the difference in SOAs. Reports of SAPEs on subsequent supraliminally presented target faces demonstrated by ERP studies using the backward masking paradigm (Pegna et al., 2008; Smith, 2011) are currently inconclusive because of the overlapping ERP responses for prime and target faces. In the present study, we chose an SOA of 300 ms because it has been used in several previous studies (Chica et al., 2014; Fazio, 2001; Folyi et al., 2019) and has been shown to be effective for facilitating the affective priming effect. Moreover, Itier and Taylor (2002) demonstrated a repetition effect on N170 amplitude, in which repetition of the same face identity suppressed the N170 response, leading to increased N170 amplitude in prime‐target incongruent conditions (e.g., upright/inverted faces with the same identity or different identities). These findings further suggest that automatic rapid encoding of emotional information included in prime stimuli is implemented in the brain and that such information is integrated with the emotional content of subsequent target stimuli by the FFA, STS, IOG, or occipital face area (OFA). From this perspective, the N170 modulation observed in the present study may indicate that females and males recruited different brain circuits when viewing fearful prime faces and subsequent target faces.

Because the presentation duration in the current study was extremely short (17 ms), it also remains unclear whether subliminal emotional properties were processed in the occipito‐temporal face‐sensitive areas. We assume that the gender differences we observed in the N170 can be partially explained by the functional connectivity between the FFA and amygdala (Pessoa et al., 2002; Vuilleumier & Pourtois, 2007). Evolutionarily, unconscious processing of threat‐related stimuli would be expected to be beneficial because it allows extremely rapid motor responses to stimuli (Costa et al., 2014). The amygdala is involved in pre‐attentive, rapid processing of threat‐related stimuli, whereby it receives subcortical visual information via the superior colliculus and pulvinar thalamus (Öhman et al., 2007), and anatomical evidence for subcortical emotional pathway connections has also been shown in nonhuman primates and humans (Tamietto & de Gelder, 2010). The sensitivity of the amygdala to emotional content has generally been investigated using a phenomenon in which subliminally presented emotional visual stimuli enhance amygdala activation (Pourtois et al., 2013; Whalen et al., 1998). A previous study reported that the amygdala appears to be involved in automatically elicited negative evaluative shifts in response to negative face priming (Suslow et al., 2013). Because the modulation of visual cortex by emotional face stimuli is dependent on the amygdala (Hadj‐Bouziane et al., 2012; Vuilleumier et al., 2004), it is possible that modulation of N170 amplitude reflects the contribution of amygdala output to the visual cortex (Pegna et al., 2008; Vuilleumier et al., 2004). Dima et al. (2011) suggested that negative emotional information is conveyed to the ventral prefrontal cortex (VPFC) via multiple pathways, including the inferior occipital gyrus, FFA, and amygdala, and is processed among these pathways in parallel. In addition, functional magnetic resonance imaging studies have reported gender differences in the processing of emotional properties, with females exhibiting enhanced activity in the amygdala in response to negative stimuli, whereas parts of the orbitofrontal cortex, anterior cingulate cortex, and dorsolateral prefrontal cortex were also reported to be recruited to a lesser extent than in males (Domes et al., 2010; Filkowski et al., 2017; Stevens & Hamann, 2012). In males, brain areas involved in cognition and cognitive control, including the prefrontal and superior parietal regions, were found to be recruited to process negative content, which was associated with increased amygdala activation (Domes et al., 2010; Filkowski et al., 2017; Koch et al., 2007). Taking these findings together, we speculate that, in females, subliminally presented fearful face stimuli in the fearful prime‐neutral target condition activate emotion‐specific areas, including the amygdala. These areas then stimulate the right occipito‐temporal region (i.e., FFA, IOG or STS) and process neutral target faces as emotionally negative (enhanced N170 amplitude). The frontal regions associated with cognitive processes may also be stimulated, but to a lesser extent than in male participants. In males, however, subliminally perceived fearful face information is conveyed to the VPFC and the right occipito‐temporal regions via the amygdala in parallel, and subsequent target faces are cognitively processed, while N170 activity is weakly moderated only by subliminal fearful faces, facilitating faster responses for neutral target faces. Another possibility is that the emotional congruency effect on the N170 in males reflects a repetition suppression effect due to the emotional familiarity between fearful prime and fearful target faces (Kouider et al., 2009; Rostami et al., 2020).

Our study involved several methodological limitations. First, the experiment lacked a control condition. Thus, we could have overlooked other SAPEs (both behavioral and electrophysiological) because we did not compare the prime condition with a primeless condition. Second, we only studied the emotion of fear. Thus, it remains unclear whether SAPEs are solely evident in the fearful face‐priming condition, and the SAPEs we observed should be interpreted with caution. In future, both subliminal priming and primeless conditions should be examined, and other types of prime and target faces should be tested (i.e., happy, sad, angry, and disgusted faces). Third, although the intensity levels for male and female face images were almost equal (see SI.1), the number of face stimuli used in our study was small. Because the ATR face database contains four female and six male actors, and because we wanted the number of male/female faces to be equal, we were limited to four female and four male face images. Fourth, considering that the mean RR for ambiguous‐fearful faces was approximately 34%, the emotional valence required to express “ambiguity” may have been insufficient (i.e., approximately 50% of the emotional valence level was required). Thus, defining and creating ambiguous‐face images should be considered in more depth. Furthermore, we did not consider individual face actors as fixed factors. Kret et al. (2011) reported that males showed more brain activity in several regions of interest, including FFA and STS, following fearful and angry male bodily expressions. Further analysis will be necessary to determine the effect of “face actor” on SAPEs.

## 5 CONCLUSIONS

In the current study, female participants exhibited increased right N170 amplitude in fearful prime trials with neutral target faces. Meanwhile, male participants exhibited decreased N170 amplitude in the right ROI in fearful prime trials with fearful target faces. Significant correlations between N170 amplitude in the fearful prime conditions and behavioral performance were observed only in male participants. Our ERP results suggest the existence of a gender difference in target‐face processing preceded by subliminally presented face stimuli in the right occipito‐temporal regions.

## CONFLICT OF INTEREST

The authors declare no conflicts of interests.

## AUTHOR CONTRIBUTIONS

MT performed the experiments and wrote the paper. MT, TM, EY, NT, and ST conceived and designed the experiments. MT and EY analyzed the data. KO, EY, and HN contributed experimental programs and analysis tools. The study was supervised by TM and ST.

### PEER REVIEW

The peer review history for this article is available at https://publons.com/publon/10.1002/brb3.2060.

## Supporting information

Supplementary Material

## Data Availability

The data set used or analyzed during the current study are available from the corresponding author on reasonable request.
