# Supplementary material for: Gender differences in subliminal affective face priming: A high‐density ERP study
Source: Brain Behav. 2021 Feb 2;11(4):e02060. doi: 10.1002/brb3.2060 (PMC8035456; doi:10.1002/brb3.2060)
Supplement: Supplementary file 1 — Supplementary Material [file BRB3-11-e02060-s001.docx]

**Supporting information**

**Gender differences in subliminal affective face priming: A high-density ERP study**

Mutsuhide Tanaka^1,2*^, Emi Yamada^1^, Toshihiko Maekawa^1^, Katsuya Ogata^1^, Naomi Takamiya^1^, Hisato Nakazono^1^, Shozo Tobimatsu^1^

^1^Department of Clinical Neurophysiology, Neurological Institute, Graduate School of Medical Sciences, Kyushu University, 3-1-1 Maidashi, Higashi-Ku, Fukuoka 812-8582, Japan

^2^Department of Occupational Therapy, School of Health Science, Kyushu University of Health and Welfare, 1714-1 Yoshino-cho, Nobeoka, 882-8508, Japan

S.1 Face stimulus data set

Images of faces were taken from the ATR face database (ATR Promotions, Inc., Kyoto, Japan). Psychological evaluation was conducted by ATR Promotions to determine the intensity of emotion for each facial image. According to their data, the average intensity of the fearful face images used in the current study was 4.49 ± 1.89 for the male actors and 4.63 ± 1.99 for the female actors (intensity level was rated from 1 to 7). These average intensity levels were almost equal, suggesting that any effect of intensity of fearful expression on gender differences would be likely to be minimal.

S.2 Definition of regions of interest

The optimal time windows for P1, N170 and P2 in females and males were determined by microstate analysis. The time windows for measuring the peak amplitudes and latencies were as follows: P1: 419–447 (119–147) ms in males and 419–447 (119–147) ms in females; N170: 444–488 (144–188) ms in males and 473–503 (173–203) ms in females; P2: 515–559 (215–259) ms in males and 511–561 (211–261) ms in females. Use of non-independent selective analysis (i.e., the use of same data for selection and selective analysis simultaneously) would result in distorted descriptive statistics and invalid statistical inference, potentially causing increased type 1 error. Thus, the electrodes for the regions of interest (ROI) were selected according to the results of previous studies (Hinojosa, Mercado, & Carretié, 2015; Rossignol, Campanella, Bissot, & Philippot, 2013; Rossion & Caharel, 2011). The ROIs for each ERP component were defined as follows: P1 and P2 (left: O1, 65, 69, 74; right: O2, 82, 89, 90), N170 (left: T5, 57, 64, 65; right: T6, 90, 95, 100).

S Figures


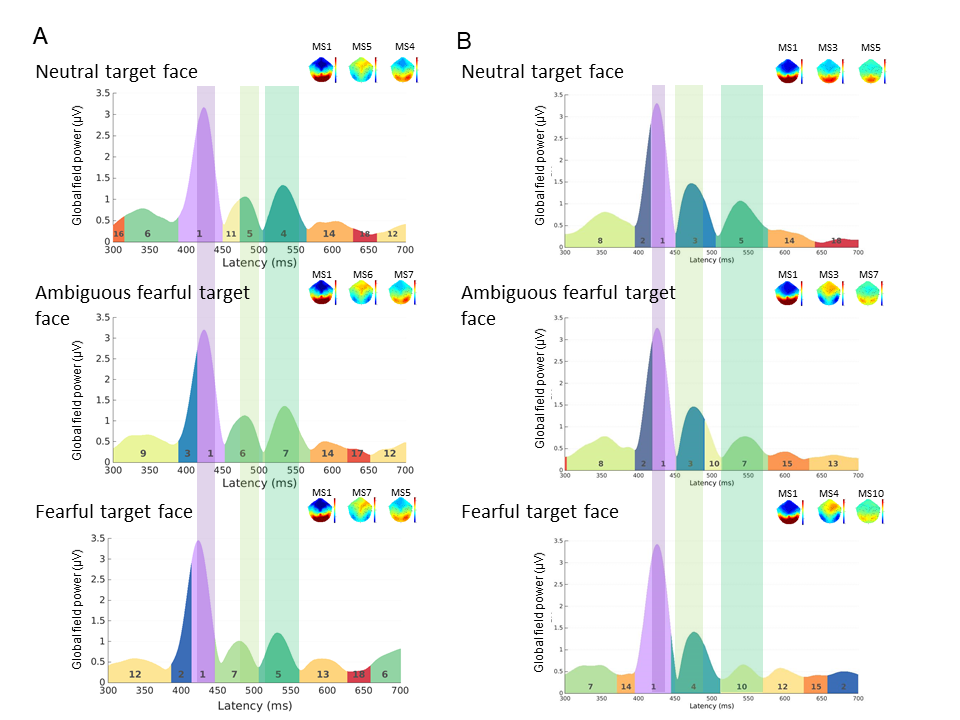


Fig. S1: Microstate analysis outlining periods of topographic differences in the grand mean ERPs in females (A) and males (B). Neutral faces were used as subliminal primes. Scalp topographies show microstate maps obtained from the cross-validation procedure. The microstate maps are displayed in the order of occurrence, from left to right. Insets show the different patterns in each condition. Red and blue indicate positive and negative potential values, respectively. In contrast to the microstate analysis for the fearful prime condition (see Fig. 3), MS5, 6, and 7, which correspond to the N170 time window in females, had almost the same duration. This suggests that subliminal priming with neutral faces did not affect ERPs in response to the subsequently presented target faces.


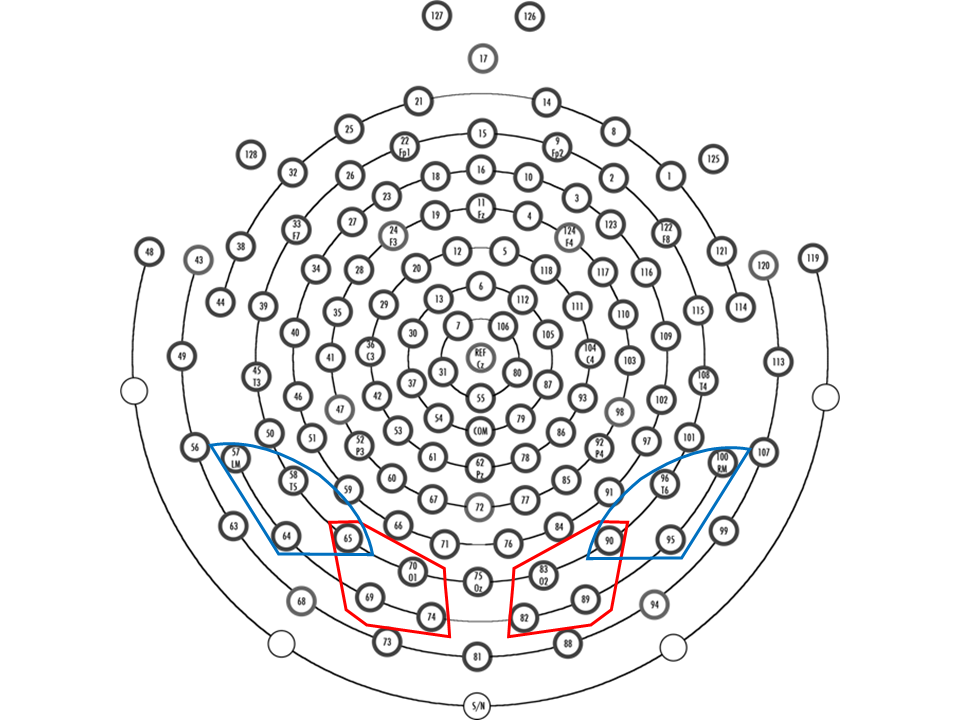


Fig. S2: Layout of the electrode array. The groups of electrodes enclosed by red borders represent the regions of interest (ROIs) for the P1 and P2 (left: O1, 65, 69, 74; right: O2, 82, 89, 90); those enclosed by blue borders indicate the ROIs for the N170 (left: T5, 57, 64, 65; right: T6, 90, 95, 100).


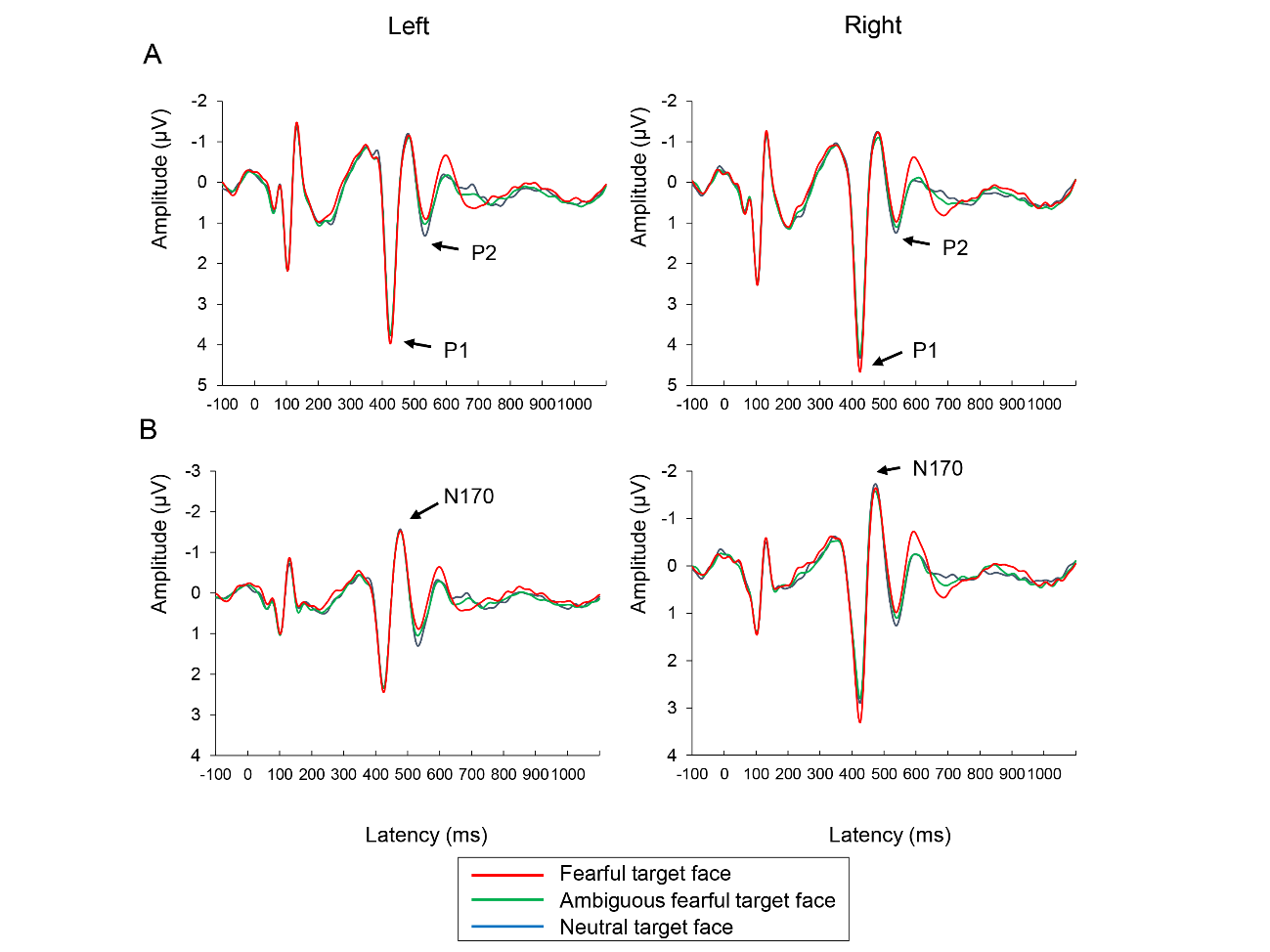


Fig. S3: Grand averaged waveforms of all participants for the P1, P2 (A), and N170 (B) for each target face in each ROI in the fearful face-prime condition. P1 amplitudes for the fearful target-face stimuli were larger than those for the neutral and ambiguous target-face stimuli in both ROIs (A). In contrast, we found no main effect of prime face, target face, or laterality for N170 amplitude (B). P2 amplitudes for the neutral target face were larger than those for the ambiguous and fearful target-face stimuli. P2 amplitudes for the ambiguous target-face stimuli were also larger than those for fearful target-face stimuli (A). Note that ERP waveforms in response to the neutral face-prime condition are not shown because of the overlapping nature of the responses.

References

Hinojosa, J. A., Mercado, F., & Carretié, L. (2015). N170 sensitivity to facial expression: A meta-analysis. *Neuroscience and Biobehavioral Reviews*, *55*, 498–509. https://doi.org/10.1016/j.neubiorev.2015.06.002

Rossignol, M., Campanella, S., Bissot, C., & Philippot, P. (2013). Fear of negative evaluation and attentional bias for facial expressions: an event-related study. *Brain and Cognition*, *82*(3), 344–352. https://doi.org/10.1016/j.bandc.2013.05.008

Rossion, B., & Caharel, S. (2011). ERP evidence for the speed of face categorization in the human brain: Disentangling the contribution of low-level visual cues from face perception. *Vision Research*, *51*(12), 1297–1311. https://doi.org/10.1016/j.visres.2011.04.003
